# Ag/TA@CNC Reinforced Hydrogel Dressing with Enhanced Adhesion and Antibacterial Activity

**DOI:** 10.3390/gels11080591

**Published:** 2025-07-31

**Authors:** Jiahao Yu, Junhao Liu, Yicheng Liu, Siqi Liu, Zichuan Su, Daxin Liang

**Affiliations:** 1 Key Laboratory of Bio-Based Material Science and Technology (Ministry of Education), Northeast Forestry University, Harbin 150040, China; 2 College of Chemistry and Chemical Engineering, China University of Petroleum (East China), Qingdao 266580, China

**Keywords:** nanocomposite hydrogel, tannic acid, cellulose nanocrystals

## Abstract

Developing multifunctional wound dressings with excellent mechanical properties, strong tissue adhesion, and efficient antibacterial activity is crucial for promoting wound healing. This study prepared a novel nanocomposite hydrogel dressing based on sodium alginate-polyacrylic acid dual crosslinking networks, incorporating tannic acid-coated cellulose nanocrystals (TA@CNC) and in-situ reduced silver nanoparticles for multifunctional enhancement. The rigid CNC framework significantly improved mechanical properties (elastic modulus of 146 kPa at 1 wt%), while TA catechol groups provided excellent adhesion (36.4 kPa to pigskin, 122% improvement over pure system) through dynamic hydrogen bonding and coordination interactions. TA served as a green reducing agent for uniform AgNPs loading, with CNC negative charges preventing particle aggregation. Antibacterial studies revealed synergistic effects between TA-induced membrane disruption and Ag^+^-triggered reactive oxygen species generation, achieving >99.5% inhibition against Staphylococcus aureus and Escherichia coli. The TA@CNC-regulated porous structure balanced swelling performance and water vapor transmission, facilitating wound exudate management and moist healing. This composite hydrogel successfully integrates mechanical toughness, tissue adhesion, antibacterial activity, and biocompatibility, providing a novel strategy for advanced wound dressing development.

## 1. Introduction

Trauma as one of the most common forms of mechanical tissue injury, is primarily caused by warfare, terrorist attacks, disasters, and accidents (including traffic accidents, lacerations, falls, and burns). The healing process is often accompanied by inflammation and infection risks. Effective treatment requires simultaneous hemostasis, promotion of tissue regeneration, and functional recovery. Wound dressings, as critical medical materials, have received increasing attention. Although traditional dressings (such as gauze and cotton) are widely used due to their strong absorption capacity, easy preparation, and low cost, they exhibit insufficient antibacterial capability and tend to adhere to newly formed granulation tissue during the healing process, leading to secondary injury upon removal [1].

With the continuous advancement of medical technology and in-depth research into wound healing mechanisms, higher performance requirements have been placed on wound dressings [2]. Of particular interest, hydrogel wound dressings are considered the most competitive advanced wound dressing materials [3]. Hydrogel dressings not only accelerate wound healing [4] but also help alleviate patient pain through wound cooling [5]. Furthermore, as wound dressings, hydrogels can absorb excess tissue exudate while providing excellent oxygen permeability [6]. Zhang et al. designed a simple one-pot photocrosslinking method to prepare multifunctional hydrogel dressings based on sodium alginate, achieving an adhesion strength of 12.5 kPa and demonstrating excellent mechanical and antibacterial properties [7]. Wang et al. prepared a polyacrylic acid-based hydrogel dressing with ideal self-healing and stretchable properties, while exhibiting strong antibacterial activity and promoting fibroblast migration among other multifunctional biological characteristics [8]. Huang et al. proposed a long-lasting hydrogel dressing NPs-PSH based on silver-catechol dynamic redox chemistry, showing good bioadhesion (14.21 kPa) and biocompatibility, achieving sustained regeneration of antioxidant catechol groups and controlled release of antibacterial Ag^+^ [9].

Silver nanoparticles (AgNPs) have been widely applied in the medical industry due to their excellent broad-spectrum antibacterial properties and low cytotoxicity [10]. Regarding AgNPs synthesis, chemically reduced AgNPs are prone to aggregation and typically require additional toxic dispersants (such as polyvinylpyrrolidone [11]), which can lead to secondary toxicity. To address these issues, the synthesis of AgNPs using green, non-toxic renewable biomaterials as reducing and stabilizing agents has attracted considerable research attention. Tannic acid (TA) is a low-cost, abundant plant-derived polyphenol that can serve as a natural reducing and stabilizing agent [12], replacing chemical reducing agents for green synthesis of silver nanoparticles. Cellulose nanocrystals (CNCs), as natural macromolecular materials, have garnered attention due to their high aspect ratio, abundant hydroxyl functional groups, and excellent biocompatibility [13]. Tannic acid forms TA@CNC complexes by coating onto rigid rod-like CNCs through hydrogen bonding [14]. The hydroxyl, carboxyl, and catechol groups on the TA@CNC surface not only enhance the mechanical properties and interfacial adhesion of covalent crosslinked networks [15], but also enable the reduction of silver ions to form AgNPs through phenolic hydroxyl groups when mixed with silver ion solutions, promoting Ag dispersion on CNCs. Moreover, the catechol groups in TA facilitate coupling with AgNPs through coordination bonds, resulting in excellent dispersion stability of AgNPs [16]. Additionally, good mechanical properties are essential characteristics for hydrogel dressings. However, natural polymer hydrogels are mechanically weak and brittle, which severely limits their applications, while hydrogels prepared from single synthetic polymers typically exhibit considerable resistance to cell adhesion. Therefore, creating composite hydrogel systems with excellent mechanical properties and biocompatibility is important yet challenging.

In this study, we designed and prepared a novel sodium alginate-polyacrylic acid-tannic acid-coated cellulose nanocrystals-silver nanoparticles [Ag/TA@CNC/SA-poly(AA)] composite hydrogel dressing. The hydrogel employs a dual crosslinking network design strategy, sodium alginate preferentially forms reversible ionic crosslinking networks through metal ions, endowing the material with flexibility and environmental adaptability; polyacrylic acid constructs stable covalent crosslinking networks via free radical polymerization, providing structural support [17]. The synergistic effect of these two crosslinking mechanisms significantly enhances the mechanical properties and tissue adhesion capability of the hydrogel. TA@CNCs serve as multifunctional nanofillers playing a crucial role: the phenolic hydroxyl groups abundant in tannic acid molecules interact with the hydrogel matrix through hydrogen bonding, enhancing the self adhesive properties of the material; the high mechanical strength and modulus of cellulose nanocrystals comprehensively improve the overall mechanical performance of the hydrogel through interfacial interactions. Regarding antibacterial functionality, the reducing properties of tannic acid are utilized to achieve in-situ reduction of silver ions, generating silver nanoparticles. Meanwhile, the negative surface charges of CNCs serve a dual stabilization role by preventing AgNP-AgNP aggregation during the in-situ reduction process on CNC surfaces and ensuring uniform dispersion of the Ag-loaded TA@CNC composites within the hydrogel matrix, thereby preventing composite aggregation and maintaining homogeneous silver distribution, thereby enhancing antibacterial efficacy. This composite hydrogel dressing successfully integrates excellent mechanical properties, strong tissue adhesion, efficient antibacterial activity, and good biocompatibility, providing a novel design approach for the development of advanced wound dressings.

## 2. Results and Discussion

### Strategy for Ag/TA@CNC/SA-Poly(AA) Nanocomposite Hydrogels

**Figure 1:**
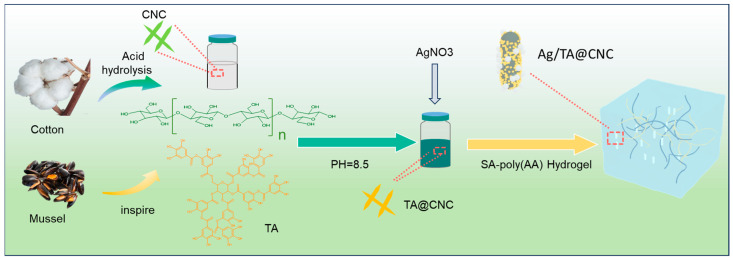
Schematic illustration of Ag/TA@CNC/SA-poly(AA) composite hydrogel preparation process.

As illustrated in Figure 1, the construction strategy of Ag/TA@CNC/SA-poly(AA) nanocomposite hydrogels centers on functionalized nanocellulose (TA@CNC) as the core reinforcing unit, combined with silver ion dispersion to achieve multifunctional hydrogels with high toughness, adhesiveness, and antibacterial properties. Initially, cellulose nanocrystals (CNCs) are prepared via acid hydrolysis [18]. The electrostatic repulsion provided by carboxyl group dissociation on CNC surfaces and their strong hydrophilicity [19] constitute the key physicochemical foundation for ensuring uniform and stable dispersion of the functionalized nanocomponent “Ag/TA@CNC” within the subsequently introduced “SA-poly(AA)” aqueous polymer matrix [20].

Tannic acid can form coatings through in-situ oxidative polymerization under alkaline conditions [21]. Under alkaline conditions, phenolic components undergo oxidation and polymerization in the presence of oxygen, forming high molecular weight structures. These macromolecules tend to accumulate on CNC surfaces due to reduced solubility, while the high affinity of CNC surfaces promotes stable adhesion of tannic acid, thereby forming robust coatings [22]. During this process, the catechol groups of TA not only endow CNC surfaces with high reactivity but also form stable interfacial bonding through hydrogen bonding and π-π stacking interactions [23]. Subsequently, TA@CNCs are dispersed in sodium alginate (SA)-acrylic acid (AA) pre-polymerization solution, where free radical polymerization is initiated by ammonium persulfate (APS) to form the SA-poly(AA) dual crosslinking network framework, involving both poly(AA) homopolymerization and potential grafting interactions as described in the literature [24]. The abundant phenolic hydroxyl groups on TA@CNC surfaces form hierarchical crosslinking networks with SA carboxyl groups and poly(AA) carboxylic acid groups through dynamic hydrogen bonding and ionic coordination. Meanwhile, the catechol groups of TA serve as metal coordination sites, achieving uniform loading of Ag nanoparticles through in-situ reduction reactions with silver ions (Ag^+^).

**Figure 2:**
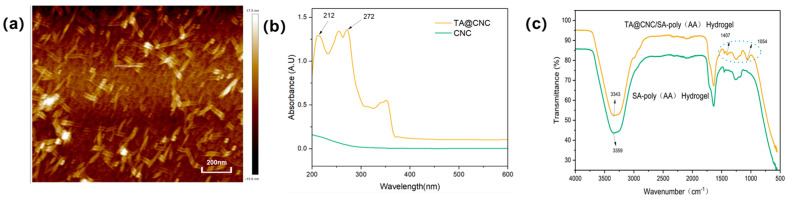
(**a**) AFM image of CNC, (**b**) UV−visible spectra of TA@CNC, (**c**) FTIR spectra of SA−poly(AA) and TA@CNC/SA-poly(AA) hydrogels.

For the characterization analysis of the prepared CNCs, atomic force microscopy (AFM) was employed to observe the samples. The AFM image (Figure 2a) clearly presents highly dispersed and size-uniform nanoscale rod-like structures with widths in the range of 10–20 nm and lengths distributed in the 100–300 nm range, which are consistent with the theoretical expected size characteristics of cellulose nanocrystals [25]. Meanwhile, no obvious aggregation phenomenon was observed in the image, indicating that CNCs successfully achieved nanoscale dispersion during the preparation process, providing strong evidence for successful CNC preparation from a microstructural perspective. The UV-vis spectrum of TA@CNC (Figure 2b) exhibits characteristic absorption peaks of TA phenolic hydroxyl groups at 212 nm and 272 nm, which are consistent with the standard TA spectrum, confirming the presence of phenolic functional groups on the surface [23]. The UV-visible spectroscopy systematically characterized the structural properties of TA@CNC.FTIR analysis (Figure 2c) revealed the integration mechanism of TA@CNC in SA-poly(AA) hydrogels. After introducing TA@CNC into the SA-poly(AA) hydrogel system, the O-H stretching vibration peak of the composite hydrogel red-shifted from 3359 cm^−1^ to 3343 cm^−1^, indicating the formation of dynamic hydrogen bonding networks between TA@CNC and the carboxylic acid groups of SA and poly(AA) chains. Simultaneously, the coordination interaction between catechol groups in TA@CNC and Ca^2+^ ions resulted in decreased intensity of the C = O peak in the composite system, with new characteristic peaks appearing at 1054 cm^−1^ and 1407 cm^−1^, attributed to -CH_2_ bending vibrations and the formation of polyphenol-metal coordination structures. In summary, spectroscopic analysis confirmed that TA@CNC was effectively embedded into the hydrogel network through multiple non-covalent interactions.

**Figure 3:**
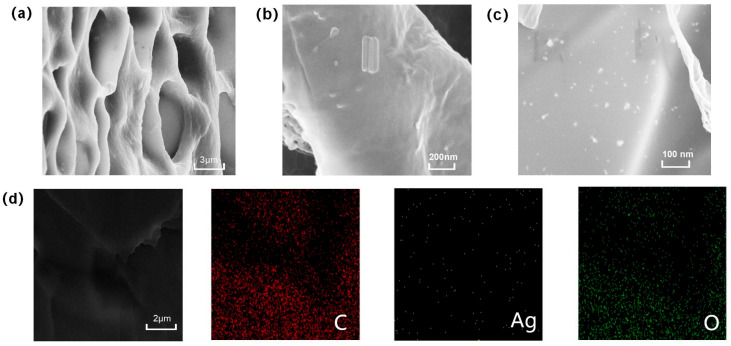
(**a**) SEM image of freeze-dried hydrogel, (**b**) SEM image of TA@CNC, (**c**) SEM image of AgNPs, (**d**) SEM-EDS elemental analysis.

Scanning electron microscopy (SEM) images reveal the microstructural characteristics of the composite hydrogel. Figure 3a shows that the freeze-dried hydrogel exhibits a three-dimensional porous structure with relatively uniform pore size distribution. Remarkably, despite the combination of two distinct crosslinking systems (AA networks and ionic SA-Ca^2+^ networks), the composite hydrogel maintains an excellent interconnected porous architecture that synergistically contributes to the enhanced mechanical modulus and functional performance. Figure 3b demonstrates that tannic acid-coated cellulose nanocrystals (TA@CNCs) are uniformly dispersed throughout the hydrogel matrix in short rod-like morphology. Figure 3c displays silver nanoparticles presenting nanoscale particulate distribution without obvious aggregation phenomena.

Further SEM-EDS combined analysis (Figure 3d) indicates that C, O, and Ag elements are uniformly distributed within the hydrogel interior, confirming the homogeneous incorporation of Ag and TA@CNCs. This confirms the uniform integration of silver nanoparticles and TA@CNC nanofillers throughout the entire hydrogel network. Such uniformity demonstrates successful composite formation, ensuring consistency in hydrogel composition and performance. The aforementioned morphological characterization results systematically reveal the hierarchical structural features of the composite material, providing morphological evidence for subsequent functional performance studies.

**Figure 4:**
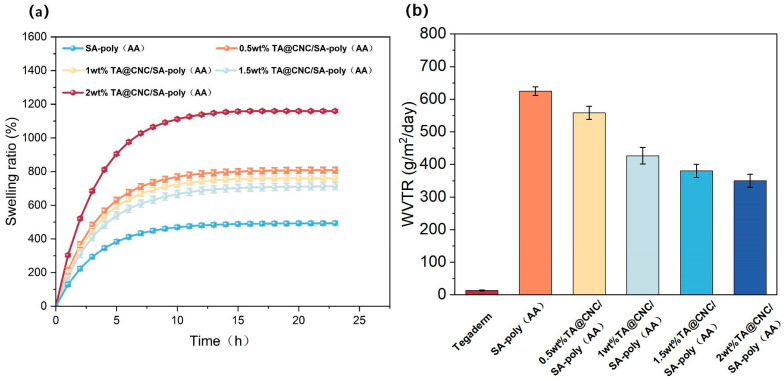
(**a**) Swelling behavior of composite hydrogels with varying TA@CNC contents, (**b**) WVTR values of Tegaderm and composite hydrogels with varying TA@CNC contents.

The TA@CNC/SA-poly(AA) nanocomposite hydrogels constructed in this study achieve precise regulation of swelling and permeability properties through multi-component synergistic effects. As shown in Figure 4a, driven by osmotic pressure mechanisms, the high osmotic pressure difference between the hydrogel network and deionized water promotes rapid water molecule penetration during the initial 9 h, resulting in fast swelling rate increase. TA@CNC concentration exhibits significant regulatory effects on swelling behavior: in the 2 wt% TA@CNC group, the three-dimensional network synergistically constructed by TA polyphenolic groups and CNC nanofibers enhances the water molecule adsorption capacity of the system, with both swelling rate and equilibrium swelling ratio superior to lower concentration groups [26]. The fundamental network formed by polyacrylic acid crosslinking, combined with the rigid CNC skeleton, ensures structural integrity of the hydrogel under high water absorption conditions.

The water vapor transmission rate (WVTR) data shown in Figure 4b indicates that although the hydrogel WVTR does not reach the ideal threshold (2000–2500 g/m^2^/d), it demonstrates significant advantages compared to the commercial dressing Tegaderm (12.23 g/m^2^/d) [27]. The pure SA-poly(AA) group exhibits peak WVTR values (approximately 600 g/m^2^/d).

After introducing TA@CNC, WVTR shows a gradient decrease with increasing concentration, attributed to the regulation of water vapor transport pathways by orderly arranged micro-nano channels and dynamic hydrogen bonding effects of TA polyphenolic groups [28]. These performance characteristics achieve dynamic balance between exudate management and gas exchange, effectively preventing bacterial proliferation and wound dehydration, providing a foundation for subsequent functional evaluations.

The TA@CNC/SA-poly(AA) composite hydrogels achieve significant optimization of mechanical properties through multi-level synergistic enhancement mechanisms, where the dual crosslinking system and introduction of TA@CNC nano-reinforcing phase exhibit excellent synergistic strengthening and toughening effects.

In tensile testing, the stress-strain behavior is distinctive. The TA@CNC nano-reinforcing phase, leveraging its high specific surface area and strong interfacial interactions, serves as physical crosslinking points and stress transfer bridges within the hydrogel network. Under external forces, it disperses stress and prevents crack propagation, enhancing toughness [29], enabling the composite hydrogel to withstand higher stress and greater tensile strain.

**Figure 5:**
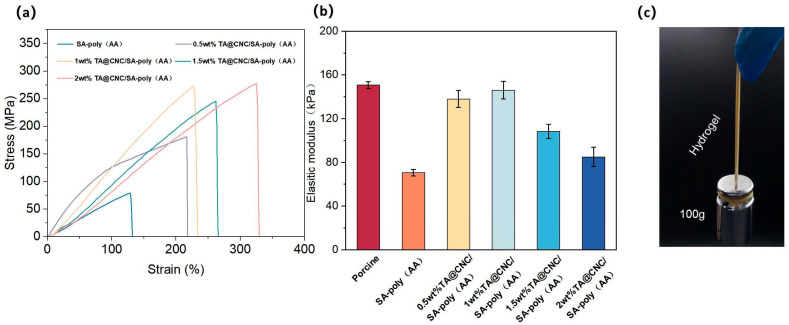
(**a**) Tensile stress-strain curves of TA@CNC/SA-poly(AA) composite hydrogels with different TA@CNC contents, (**b**) Elastic modulus of composite hydrogels with varying TA@CNC contents, (**c**) Digital photographs of the hydrogel lifting 100 g weight.

From the stress-strain curves in Figure 5a, as TA@CNC nanofiller content increases (0.5–2 wt%), significant differences in mechanical response compared to SA-poly(AA) are observed. The nanoparticles optimize stress bearing and strain adaptability. Low content shows limited enhancement effects, while 1–1.5 wt% exhibits optimal synergy. Excessive content causes aggregation that disrupts network uniformity.

The elastic modulus data in Figure 5b confirms that appropriate content (around 1 wt%) enables the composite hydrogel modulus to approach that of human skin (corresponding to porcine data [30]). Through dual crosslinking network synergy, the tensile mechanical properties are optimized, establishing a solid foundation for biomedical applications such as skin repair. Hydrogels with Young’s modulus matching biological tissues are highly suitable for skin adhesion [31], reducing discomfort and tissue damage risks. Figure 5c demonstrate the mechanical strength through digital photographs of the hydrogel lifting 100 g weight, visually confirming the enhanced load-bearing capacity.

**Figure 6:**
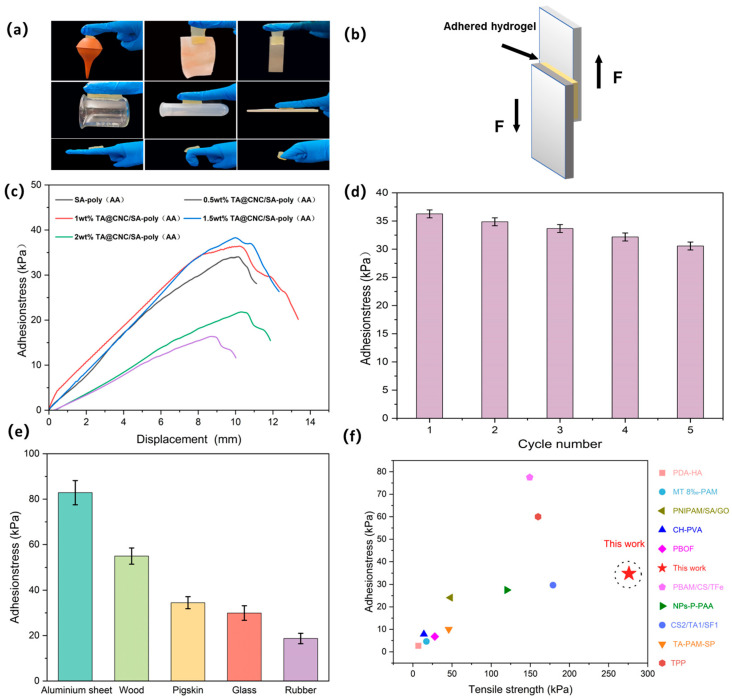
(**a**) TA@CNC/SA-poly(AA) hydrogel adhered to various material surfaces, (**b**) Scheme oflap shear test geometry, (**c**) Adhesion tensile curves and adhesion strength of TA@CNC/SA-poly(AA) hydrogels at different TA@CNC contents, (**d**) Cyclic adhesion performance, (**e**) Adhesion strength of TA@CNC/SA-poly(AA) hydrogel to aluminum sheet, wood, pigskin, glass, and rubber, (**f**) Comparison of adhesion and tensile strength in this work with previous reports.

The TA@CNC/SA-poly(AA) composite hydrogels demonstrate excellent universal adhesion properties. Experimental results show that the hydrogel can firmly adhere to both dry hydrophilic substrates (such as glass and metal) and hydrophobic substrates (such as Teflon and plastic). When applied to human skin, the hydrogel can withstand dynamic deformation from finger bending (0° to 135°) and leaves no residue upon removal, meeting the dual requirements of interface compatibility and mechanical reliability for biological dressings. As illustrated in Figure 6a, the adhesion mechanism originates from multi-component synergistic effects: carboxylic acid groups (-COOH) of acrylic acid (AA) anchor to polar surfaces (such as skin and rubber) through hydrogen bonding and ion-dipole interactions; catechol groups of tannic acid (TA) adhere to non-polar substrates (such as polytetrafluoroethylene) through hydrophobic interactions and π-π stacking; additionally, the nanofiber structure of CNCs embeds into substrate surface microstructures through mechanical interlocking effects, significantly enhancing effective contact area. Quantitative analysis through lap shear testing (Figure 6b,c) reveals that 1 wt% TA@CNC/SA-poly(AA) achieves an adhesion strength of 36.4 kPa to pigskin, representing a 122% improvement over the pure SA/AA system (16.37 kPa).Additionally, cyclic adhesion testing demonstrated that the hydrogel retains approximately 85% of its initial adhesion strength after 10 application/removal cycles on porcine skin (Figure 6d), indicating good adhesive durability for practical applications. Comparison of adhesion performance on different substrates (Figure 6e) shows adhesion strengths of 82.85 kPa, 54.93 kPa, 29.93 kPa, and 18.72 kPa for aluminum sheet, wood, glass, and rubber, respectively.

This characteristic is attributed to functional complementarity between components: TA@CNCs balance adhesion and cohesion through dynamic hydrogen bonding (TA-substrate) and rigid nano-reinforcement (CNCs); the covalent crosslinking network of AA and ionic network of SA synergistically resist shear stress; while SA-Ca^2+^ coordination bonds further strengthen interfacial bonding with metallic substrates. Figure 6f presents a comparison of adhesion and tensile strength in this work with previous reports. Compared to other hydrogels reported in previous studies, the TA@CNC/SA-poly(AA) hydrogel exhibits superior adhesion force and mechanical strength, demonstrating simultaneous optimization of adhesion strength and tensile. strength [32,33,34,35,36,37,38,39,40].

**Figure 7:**
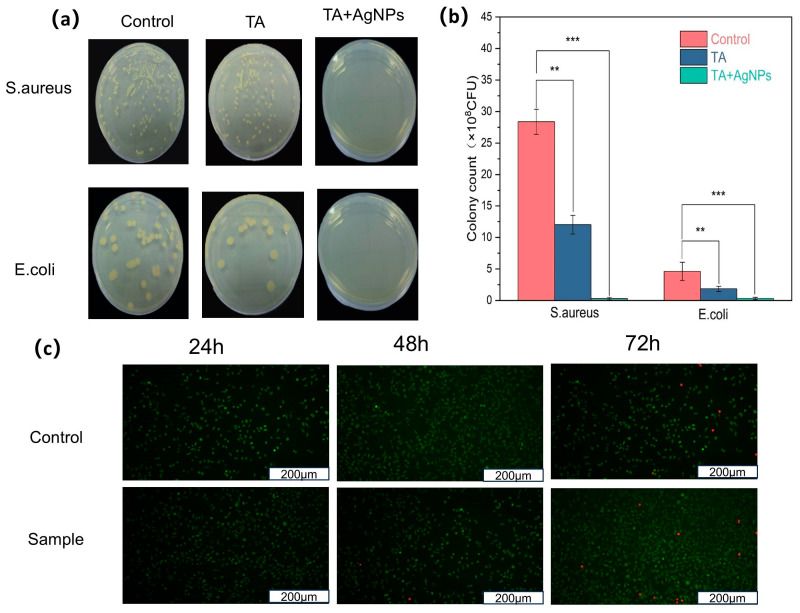
(**a**) Typical morphologies of *S. aureus* and *E. coli* colonies after 24 h culture on LB plates, (**b**) CFU counts of *S. aureus* and *E. coli* after treatment with Ag/TA@CNC/SA-poly(AA) hydrogel and TA@CNC/SA-poly(AA) hydrogel. Mean values and error bars represent mean ± SD; ** *p* < 0.01, *** *p* < 0.001, (**c**) Live/dead staining of NIH/3T3 cells after 24, 48, and 72 h incubation with Ag/TA@CNC/SA-poly(AA) hydrogel. Green: living cells (calcein-AM); Red: dead cells (EthD-1). Scale bar = 100 μm.

The physical property characterization was primarily conducted on TA@CNC/SA-poly(AA) formulations, as preliminary studies indicated that AgNP incorporation has minimal impact on the fundamental hydrogel properties (Supplementary Figures S2 and S3). The bacterial colony growth phenotypes in Figure 7a and colony counting results in Figure 7b demonstrate that TA@CNC/SA-poly(AA) hydrogel exhibits limited antibacterial effects, while the Ag/TA@CNC/SA-poly(AA) composite hydrogel shows highly efficient antibacterial activity against Staphylococcus aureus (ATCC 6538) and Escherichia coli (ATCC 25922), achieving a bacterial inhibition rate of 99.5%.Antibacterial mechanism: The antibacterial mechanism originates from the synergistic effects of tannic acid (TA) and silver nanoparticles (AgNPs). TA can bind to lipids in cell membranes and disrupt them, leading to leakage of intracellular components and ultimately cell death [41]. Additionally, TA can bind to certain enzymes in microorganisms and inhibit their activity, thereby suppressing growth and reproduction [42]. The polyphenolic groups of TA disrupt the conformational stability of bacterial membrane proteins through chelation effects, inducing surface protein crosslinking and precipitation.

Released AgNPs disrupt cell membranes, causing leakage of intracellular materials and resulting in bacterial death [43]. AgNPs may penetrate cell membranes and directly enter the cell interior, destroying internal cellular structures [44]. Consequently, cellular DNA and proteins are damaged, ultimately leading to bacterial death [45]. Ag^+^ released from AgNPs interferes with microbial electron transport chains, triggering reactive oxygen species (ROS) bursts [46], causing DNA damage and ribosome inactivation. Synergistic effects: TA-induced bacterial membrane disruption facilitates intracellular penetration of AgNPs/Ag^+^, while Ag^+^-induced enhanced membrane permeability improves TA’s targeting effects on membrane proteins. This synergistic interaction forms an efficient antibacterial system, providing effective infection control for wound dressing applications. The sustained antibacterial activity is supported by controlled release kinetics of both active components. Release studies conducted in PBS (pH 7.4) at 37 °C demonstrate sustained release of TA and Ag^+^ over 7 days (Figure S4), with TA showing initial burst release followed by steady sustained release, while Ag^+^ exhibits more gradual release kinetics. This controlled release profile ensures prolonged antimicrobial protection while minimizing potential cytotoxicity.

The biocompatibility of the Ag/TA@CNC/SA-poly(AA) hydrogel was evaluated using NIH/3T3 mouse embryonic fibroblast cells through live/dead fluorescence staining assay. As demonstrated in Figure 7c, the hydrogel exhibited excellent cytocompatibility across all tested time points (24, 48, and 72 h). The live/dead staining results show predominantly green fluorescence from viable cells with minimal red fluorescence from dead cells, indicating that the hydrogel does not release harmful substances that could compromise cell viability. The cell morphology remained normal throughout the incubation period, with cells maintaining their characteristic spindle-shaped appearance and forming confluent monolayers. These results confirm that the incorporation of silver nanoparticles and tannic acid-coated cellulose nanocrystals does not induce significant cytotoxicity, making the composite hydrogel suitable for wound healing applications where direct contact with living tissues is inevitable.

## 3. Conclusions

This study successfully constructed a nanocomposite hydrogel dressing based on TA@CNC/SA-polyacrylic acid dual networks, achieving multifunctional synergistic enhancement through incorporation of in-situ reduced silver nanoparticles (AgNPs). The material innovatively exploits the multiple functions of TA@CNC: the rigid CNC framework serves as a nano-reinforcing phase, significantly enhancing mechanical properties; catechol groups of TA provide universal adhesion capability through dynamic hydrogen bonding and coordination interactions; simultaneously, TA acts as a green reducing agent for in-situ uniform loading of AgNPs, while CNC electrostatic repulsion inhibits particle aggregation. Regarding antibacterial mechanisms, TA-induced bacterial membrane disruption and Ag^+^-triggered ROS generation form synergistic effects, achieving >99.5% inhibition rates against Staphylococcus aureus and Escherichia coli. Additionally, the TA@CNC-regulated three-dimensional porous structure balances swelling performance and water vapor transmission rate, effectively managing wound exudate while maintaining a moist healing environment. This dressing integrates biocompatibility, mechanical toughness, and efficient antibacterial activity, providing a novel strategy for wound repair material design. The material demonstrates promising potential for advanced wound care applications, though further in vivo studies are needed to validate its healing-promoting efficacy and clinical translation potential.

## 4. Materials and Methods

### 4.1. Materials

The reagents used in this experiment included acrylic acid (AA, analytical grade), ammonium persulfate (APS, analytical grade), sodium alginate (SA, Mn = 357,475, M/G molar ratio = 1.32), calcium chloride (CaCl_2_, analytical grade), silver nitrate (AgNO_3_, analytical grade), tannic acid (TA, chemical grade), and N,N′-methylenebisacrylamide (MBA, chemical grade). All reagents were purchased from commercial suppliers such as Sigma-Aldrich of Shanghai China. All chemicals were used as received without further purification. Deionized water was used throughout the experiments.

### 4.2. Preparation of Cellulose Nanocrystals (CNCs)

Cotton fibers were cut into small pieces and mechanically ground. Accurately weighed cotton fiber powder (10 g) was mixed with deionized water (106 g) and concentrated sulfuric acid (98%, 201 g). The mixture was pre-treated under mechanical stirring at 500 rpm for 15 min, then transferred to a constant temperature water bath at 45 °C and maintained at 500 rpm for continuous reaction for 140 min. The reaction was terminated by adding 5 times the mass of deionized water. After settling and phase separation, the mixture was centrifuged at 1000 rpm for 10 min. This operation was repeated until the supernatant exhibited a milky appearance, yielding CNC suspension. The suspension was placed in dialysis tubing and dialyzed against deionized water, which was changed every 4 h under magnetic stirring at 600 rpm. The neutralized suspension was then ultrasonicated to achieve nanofiber dispersion. Finally, the suspension was concentrated to the target concentration using a rotary evaporator (58 °C water bath, 190 rpm) and stored at 4 °C for future use [47].

### 4.3. Preparation of Modified CNCs (TA@CNC and Ag/TA@CNC)

The prepared CNC suspension (1 wt%) was mixed with tannic acid (TA) at a mass ratio of 10:1 under vigorous stirring and ultrasonication to ensure uniform mixing. Tris buffer solution was then added to the resulting suspension to adjust the pH to 8.5. After stirring at room temperature for 24 h, the suspension color changed from white to dark green. The product was purified by repeated centrifugation at 9000 rpm and washing with deionized water several times to obtain TA@CNC suspension, which was concentrated to 1 wt% for the next step. For further modification, silver nitrate solution (0.1 M) was added to the above TA@CNC suspension (1 wt%) and the mixture was stirred at room temperature for 6 h. The product (Ag/TA@CNC) was collected by centrifugation at 9000 rpm and washed with deionized water several times.

### 4.4. Preparation of the Nanocomposite Hydrogels

TA@CNC/SA-poly(AA) hydrogels with different TA@CNC contents were prepared as follows. The reaction was conducted in a 50 mL glass beaker placed in a temperature-controlled water bath equipped with magnetic stirring. Sodium alginate (SA, 0.300 g) was dissolved in deionized water (20 mL) by heating at 60 °C. After natural cooling to room temperature, MBA solution (0.04 g in 10 mL deionized water) was added dropwise under continuous stirring (500 rpm). Subsequently, acrylic acid (AA, 3.6 g, 0.05 mol), APS (0.03 g dissolved in 10 mL deionized water), and CaCl_2_ solution (0.05 g dissolved in 10 mL deionized water) were added. Finally, different volumes of TA@CNC suspension were added to the above solution, followed by homogeneous stirring for 60 min at ambient temperature and reaction at 60 °C for 4 h to obtain TA@CNC/SA-poly(AA) hydrogels. The hydrogels were rinsed with deionized water and gently surface-wiped repeatedly to remove any surface residues before characterization. The TA@CNC contents in TA@CNC/SA-poly(AA) hydrogels were 0.0 wt%, 0.5 wt%, 1.0 wt%, 1.5 wt%, and 2.0 wt% (based on the weight of added AA), designated as SA-poly(AA), 0.5 wt% TA@CNC/SA-poly(AA), 1.0 wt% TA@CNC/SA-poly(AA), 1.5 wt% TA@CNC/SA-poly(AA), and 2.0 wt% TA@CNC/SA-poly(AA) hydrogels, respectively. Further increasing TA@CNC content led to flocculation formation, possibly due to nanofiller aggregation. Ag/TA@CNC/SA-poly(AA) hydrogels were prepared using a similar method to TA@CNC/SA-poly(AA) hydrogels.

### 4.5. Atomic Force Microscopy (AFM) Analysis

A drop of CNC dispersion was placed on the surface of a mica sheet for air drying. The morphology of the sample was tested with a scanning area of 5.0 μm × 5.0 μm (JSPM-5200, JEOL, Tokyo, Japan).

### 4.6. UV–Visible Spectroscopy Analysis

UV–Visible spectroscopy was used to confirm the presence of tannic acid coating on the surface of cellulose nanocrystals. A stock solution of tannic acid (200 ppm) was prepared in Milli-Q water at pH 7. All samples were diluted to a concentration of 0.1 wt% using Milli-Q water and probe sonicated for 2 min prior to measurement.

### 4.7. Fourier Transform Infrared (FTIR) Analysis

FTIR spectra of TA@CNC/SA-poly(AA) hydrogels were determined with an FTIR spectrometer (Thermo Fisher Scientific, Waltham, MA, USA). First, the freeze-dried samples were thoroughly ground and mixed with dry potassium bromide at a weight ratio of 1:100. Second, the mixed powder was pressed into tablets and placed into the instrument for scanning. Finally, the data were obtained by using the FTIR spectrometer from 4000 to 400 cm^–1^ at a resolution of 4 cm^–1.^

### 4.8. Scanning Electron Microscopy—Energy Dispersive X-Ray Spectroscopy (SEM-EDS)

The microscopic morphologies of hydrogels were characterized using cold field scanning electron microscopy (SEM, HITACHI S-4800, Hitachi High-Technologies, Tokyo, Japan) coupled with energy-dispersive X-ray spectroscopy (EDS, Bruker QUANTAX 70, Bruker Corporation, Berlin, Germany). Samples were sectioned into thin strips, adhered to SEM disks with conductive glue, and rapidly frozen in liquid nitrogen prior to freeze-drying. Sublimation of ice crystals was conducted at −80 °C for 50 min in a cryostat chamber, followed by gold sputtering before transferring to the microscope chamber maintained at −150 °C for ultrastructural observation. For elemental analysis, cryogenically fractured surfaces of lyophilized hydrogels were examined using a tabletop SEM (Hitachi TM 7000, Hitachi High-Technologie, Tokyo, Japan), with EDS mapping employed to investigate the spatial distribution of silver nanoparticles (AgNPs) across the hydrogel matrix. This integrated approach enabled simultaneous topographical evaluation and elemental composition analysis under optimized cryogenic conditions to preserve the native hydrogel architecture.

### 4.9. Water Vapor Transmission Rate (WVTR) Analysis

The WVTR of TA@CNC/SA-poly(AA)hydrogels was measured according to a previous study with a slight modification [48]. Measurements were performed on three independent samples (*n* = 3) for each formulation. Briefly, dry anhydrous calcium chloride (3.0 g) was placed in a 2.5 mm diameter evaporating dish and sealed with the hydrogels. Then, the evaporating dishes were placed in an environment with a relative humidity of 75% at room temperature. The dishes were taken out after 24 h and weighed again. The *WVTR* was calculated by the following formula:

(1) $$WVTR(\frac{g}{m^{2}}\text{·}\mathrm{day})\;=\;\frac{W_{t}-W_{0}}{A}$$

where *A* is the area of the evaporating dish. *W_t_* and *W*_0_ are the initial weight and that after 24 h, respectively.

### 4.10. Swelling Properties of the Hydrogels

To analyze the swelling behaviors of the wound dressings, three replicate hydrogel samples (*n* = 3) for each TA@CNC concentration with a radius of 20 mm and a thickness of 3 mm were assessed in distilled water at 25 °C. Continuous monitoring over 23 h was achieved through a collaborative approach involving three researchers working in 8-h rotating shifts to ensure consistent measurements throughout the entire period, including overnight hours.

Measurements were taken every hour by carefully removing samples from the water bath. After the weight being equilibrium, the swollen hydrogel samples were gently blotted with filter paper to remove surface water and weighed immediately. The swelling ratios (*G*) were defined as:

(2) $$G(\%)\;=\;\frac{M_{1}-M_{0}}{M_{0}}\;\text{×}\;100$$

where *M*_0_ is the initial weight of hydrogels and *M*_1_ is the weight of the hydrogels after absorbing water [49].

### 4.11. Mechanical Properties of TA@CNC/SA-poly(AA) Hydrogels

The tensile mechanical performance of TA@CNC/SA-poly(AA) hydrogels was evaluated by a universal tensile machine (Brookfield CT3, St. Louis, MO, USA) equipped with a 100 N load. The tensile stress–strain data were recorded by extending hydrogel strips (15 × 40 × 5 mm) with a constant speed of 10 mm/min at room temperature.

### 4.12. Adhesive Strength of TA@CNC/SA-poly(AA) Hydrogels

The adhesive properties of TA@CNC/SA-poly(AA) hydrogels were evaluated by using the lap-shear test at room temperature on multiple substrate materials including porcine skin, aluminum sheet, wood, glass, and rubber. For porcine skin testing, degreased porcine skin tissue was cut into long rectangular strips (50 × 10 × 5 mm), while other substrate materials (aluminum sheet, wood, glass, and rubber) were prepared as test substrates and cleaned with ethanol to remove any surface contaminants, with their exact dimensions not strictly constrained as long as they provided sufficient area for testing. The hydrogels adhered between two pieces of the respective substrate material, and the adhesive area was consistently maintained at 10 × 10 × 5 mm for all tests regardless of substrate type. Then, the adhesive strength was measured by the universal tensile machine (Brookfield CT3, USA) at a speed of 5 mm/min until two pieces of substrate material were separated. The adhesive strength was then calculated with the following formula: where *F* refers to the maximum load of adhesion and *A* in this study.

(3) $$adhesive\;strength=\frac{F}{A}$$

represents the sample’s overlapping area [50], which is 1 cm^2^.

### 4.13. Antibacterial Activity of the TA@CNC/SA-poly(AA) Hydrogels and Ag/TA@CNC/SA-poly(AA) Hydrogels

The antibacterial activities of Hydrogels were determined by colony assay method. The bacterial of *E. coli* (ATCC 25922) and *S. aureus* (MCCCB 26003) were used to evaluate the antibacterial effect of the dressings, respectively. For quantitative evaluation, 10^8^ CFU/mL of *E. coli* and *S. aureus* (500 μL) were transferred into sterilized Luria-Bertani broth (LB) medium (30 mL). Prior to antibacterial testing, hydrogel samples were wrapped in aluminum foil and sterilized in an autoclave to ensure sterility. Then the sterilized hydrogels (5 g) were soaked in the above LB medium respectively and incubated in a shaker with 120 rpm at 37 °C for 24 h. After being diluted to be 10^−5^ times of the original bacterial solution concentration, these bacterial suspensions (10 μL) were pread evenly onto the solid surfaces of the medium. The number of the colony-forming units (CFUs) was counted after being incubated at 37 °C (*E. coli* and *S. aureus*) for 24 h, respectively, and each colony assay was repeated for more than three independent times. Bacterial suspensions without hydrogels were used as controls.

### 4.14. Cytotoxicity Assay

The in vitro cytotoxicity of the Ag/TA@CNC/SA-poly(AA) hydrogel was evaluated using NIH/3T3 mouse embryonic fibroblast cell line (ATCC) as a model system. Cells were cultured in Dulbecco’s Modified Eagle Medium (DMEM) supplemented with 10% fetal bovine serum and 1% penicillin/streptomycin at a density of 1 × 10^4^ cells mL^−1^. The culture was maintained in a humidified incubator at 37 °C with 5% CO_2_ atmosphere.

Hydrogel samples were cut into circular discs with a diameter of 1 cm and pre-soaked in culture medium overnight before cell seeding. Cells were then seeded in 24-well culture plates and incubated for 24, 48, and 72 h, respectively. After each incubation period, the culture medium was removed, and the cells were stained with a live/dead fluorescence kit containing 2 μM calcein-AM and 8 μM ethidium bromide (EthD-1) in phosphate-buffered saline. After 30 min of incubation at 37 °C, cell viability was observed under a fluorescence microscope. Living cells exhibited green fluorescence due to calcein-AM, while dead cells showed red fluorescence from ethidium bromide. Images were captured using a fluorescence microscope equipped with appropriate filter sets for green (excitation/emission: 494/517 nm) and red fluorescence (excitation/emission: 528/617 nm).

### 4.15. Sustained Release Profile

Release kinetics of TA and Ag^+^ were evaluated by immersing hydrogel samples (1 cm diameter) in PBS (pH 7.4) at 37 °C. At predetermined time intervals over 7 days, aliquots were withdrawn and analyzed by UV-vis spectroscopy (TA at 276 nm) and ICP-OES (Ag^+^). All experiments were performed in triplicate (*n* = 3).

## Data Availability

The data presented in this study are available on request from the corresponding authors.

## Supplementary Materials

The following supporting information can be downloaded at: https://www.mdpi.com/article/10.3390/gels11080591/s1, Figure S1: (a) Comparison of dispersion of nanosilver in solution with and without CNC: (b) nanosilver is uniformly dispersed after CNC addition, and (c) nanosilver tends to agglomerate without CNC addition; Figure S2: (a) Influence of nano silver on the swelling rate of hydrogel (b) Influence of nano silver on the gas permeability of hydrogel; Figure S3: (a) Effect of nano silver on the strain of hydrogel (b) Effect of nano silver on the stress of hydrogel (c) Effect of nano silver on the adhesion of hydrogel; Figure S4: Release data of tannic acid and silver ions over a one-week period.
