# Supplementary material for: Ag/TA@CNC Reinforced Hydrogel Dressing with Enhanced Adhesion and Antibacterial Activity
Source: Gels. 2025 Jul 31;11(8):591. doi: 10.3390/gels11080591 (PMC12385837; doi:10.3390/gels11080591)
Supplement: Supplementary file 1 [file gels-11-00591-s001.zip › gels-3765067-supplementary.pdf]

Supporting Information

Ag/TA@CNC Reinforced Hydrogel Dressing with Enhanced Adhesion andAntibacterial Activity

1.Dispersion of nano silver

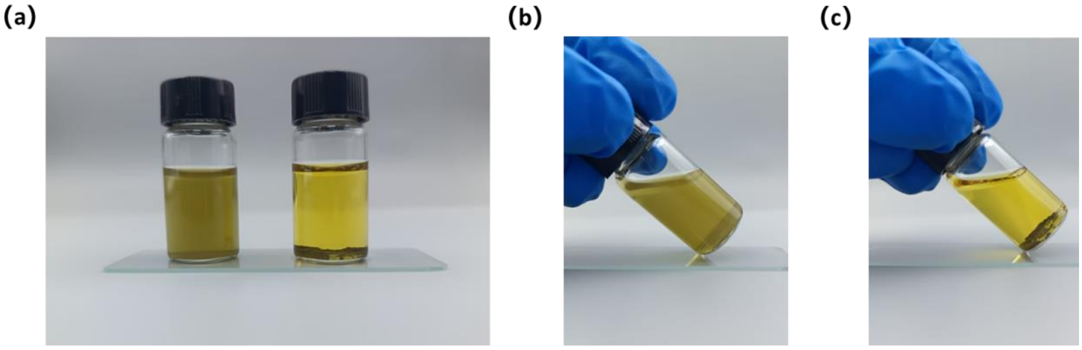

.

Figure S1. (a) Comparison of dispersion of nanosilver in solution with and without CNC: (b) nanosilver is uniformly dispersed after CNC addition, and (c) nanosilver tends to agglomerate without CNC addition.

2. The influence of nano silver on the swelling rate and gas permeability of hydrogel

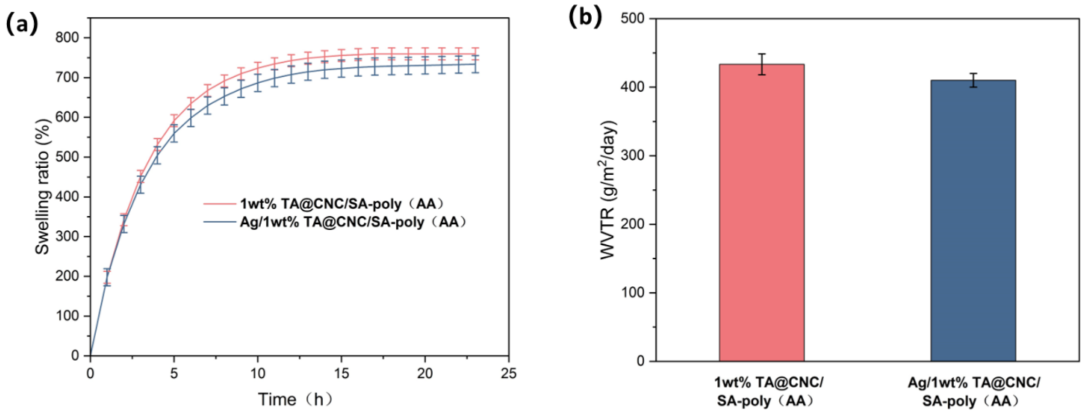

Figure S2. (a) Influence of nano silver on the swelling rate of hydrogel (b) Influence of nano silver on the gas permeability of hydrogel

3.Effect of nano silver on stress, strain and adhesion of hydrogels

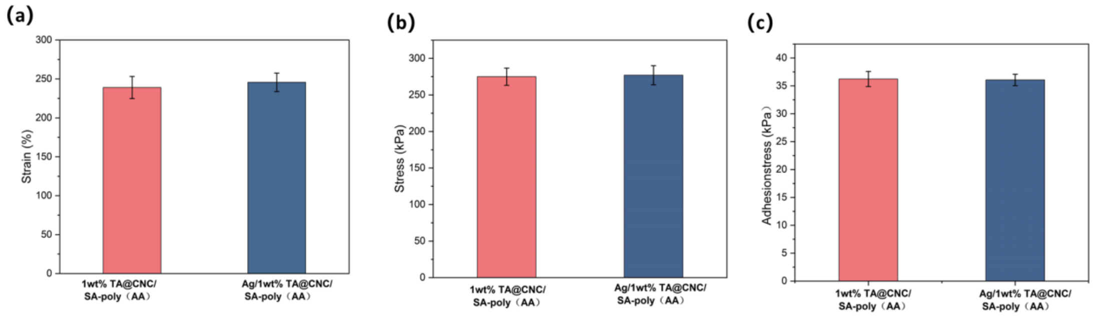

Figure S3.(a) Effect of nano silver on the strain of hydrogel (b) Effect of nano silver on the stress of hydrogel (c) Effect of nano silver on the adhesion of hydrogel

4.The Release Percentage and Fluctuation Pattern of Tannic Acid and Silver Ions over a One-Week Period

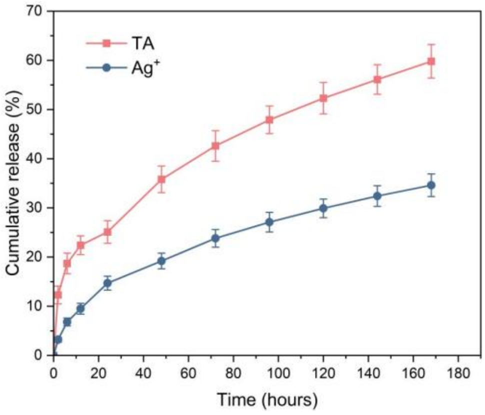

Figure S4.：Release data of tannic acid and silver ions over a one-week period
